# Selective postsaccadic enhancement of motion perception

**DOI:** 10.1016/j.visres.2021.06.011

**Published:** 2021-07-17

**Authors:** Adela S.Y. Park, Alexander C. Schütz

**Affiliations:** a Experimental and Biological Psychology, University of Marburg, Marburg, Germany; b Center for Mind, Brain and Behavior, University of Marburg, Marburg, Germany

**Keywords:** Postsaccadic enhancement, Motion discrimination, Centre-surround antagonism, Spatial suppression

## Abstract

Saccadic eye movements can drastically affect motion perception: during saccades, the stationary surround is swept rapidly across the retina and contrast sensitivity is suppressed. However, after saccades, contrast sensitivity is enhanced for color and high-spatial frequency stimuli and reflexive tracking movements known as ocular following responses (OFR) are enhanced in response to large field motion. Additionally, OFR and postsaccadic enhancement of neural activity in primate motion processing areas are well correlated. It is not yet known how this postsaccadic enhancement arises. Therefore, we tested if the enhancement can be explained by changes in the balance of centre-surround antagonism in motion processing, where spatial summation is favoured at low contrasts and surround suppression is favoured at high contrasts. We found motion perception was selectively enhanced immediately after saccades for high spatial frequency stimuli, consistent with previously reported selective postsaccadic enhancement of contrast sensitivity for flashed high spatial frequency stimuli. The observed enhancement was also associated with changes in spatial summation and suppression, as well as contrast facilitation and inhibition, suggesting that motion processing is augmented to maximise visual perception immediately after saccades. The results highlight that spatial and contrast properties of underlying neural mechanisms for motion processing can be affected by an antecedent saccade for highly detailed stimuli and are in line with studies that show behavioural and neuronal enhancement of motion processing in non-human primates.

## 1 Introduction

Functional differences between central and peripheral vision require objects of interest to be placed on the fovea. Rapid, ballistic eye movements known as saccades reposition our gaze as we navigate our visual environment. While saccades allow for the inspection of fine details, they also pose challenges for the visual system because they dissociate object position and motion in external space from image position and motion on the retina. Together with the resulting rapid, large-field motion of the stationary surround on the retina, saccades have the potential to severely disrupt visual perception. Due to a number of different factors, contrast sensitivity is strongly suppressed during saccades. While this saccadic suppression of contrast sensitivity has been studied in great detail in psychophysical and neurophysiological studies (for reviews see Matin, 1974; Volkmann, 1986; Ibbotson, Crowder, Cloherty, Price, & Mustari, 2008; Wurtz, 2008; Binda & Morrone, 2018), comparatively few studies have investigated positive modulations of visual perception directly after saccades. The postsaccadic period is highly important because the visual scene changes markedly during saccades, and because one can assume that postsaccadic vision is directed at the object that is deemed as most relevant by the organism (for reviews see Schütz, Braun, & Gegenfurtner, 2011; Tatler, Hayhoe, Land, & Ballard, 2011; Hayhoe, 2017). For instance, rapid information uptake after a saccade is crucial to minimize fixation durations and indeed, information sampling can start immediately after saccade offset (Wolf & Schütz, 2015).

Postsaccadic enhancement of contrast sensitivity has been demonstrated in humans (Burr, Morrone, & Ross, 1994; Knoll, Binda, Morrone, & Bremmer, 2011; Dorr & Bex, 2013). The spatiotemporal profile of perisaccadic contrast sensitivity shows suppression just before and during saccades for low spatial frequency luminance contrast. However, sensitivity for high spatial frequency luminance and chromatic contrast is unimpaired perisaccadically and even enhanced postsaccadically (Burr et al., 1994; Knoll et al., 2011; Dorr & Bex, 2013). This selective postsaccadic enhancement of contrast sensitivity for high spatial frequency luminance and isoluminant stimuli starts 50–100 ms after saccade onset and persists until around 300 ms after saccade onset. Interestingly, Knoll et al. (2011) showed that the postsaccadic enhancement was not specifically linked to the execution of saccades, as the enhancement was also observed following saccade-like displacements of the retinal image. However, under more naturalistic conditions, Dorr and Bex (2013) demonstrated enhancement of contrast sensitivity only under active (free viewing), compared to passive (fixation) conditions, when the retinal input was matched. These results provide direct evidence of perceptual changes resulting from increased sensitivity at the saccade endpoint, although it remains unknown how this is achieved.

Even stronger postsaccadic modulations have been reported in oculomotor and neurophysiological studies. Single-unit recordings in primates of brain areas involved in visual processing, such as in the lateral geniculate nucleus (LGN) (Reppas, Usrey, & Reid, 2002), primary visual cortex (V1) (Kagan, Gur, & Snodderly, 2008; Rajkai et al., 2008), and parietal cortex (medial temporal (MT) and medial superior temporal (MST) areas) (Ibbotson, Price, Crowder, Ono, & Mustari, 2007; Bremmer, Kubischik, Hoffmann, & Krekelberg, 2009; Cloherty, Mustari, Rosa, & Ibbotson, 2010) show enhancement that starts immediately after a saccade and lasts for several hundred milliseconds. Latencies of vergence eye movements in response to disparity signals are reduced following a saccade (Busettini, Miles, & Krauzlis, 1996). Additionally, smooth tracking, reflexive eye movements in response to large-field motion are associated with shorter latencies and initial eye speeds that are 3–4 times larger when preceded by a saccadic eye movement (Kawano & Miles, 1986). This reflexive eye movement, known as the ocular following response (OFR), is well correlated with the post-saccadic neuronal enhancement in motion processing areas of the primate (Kawano & Miles, 1986; Takemura & Kawano, 2006; Ibbotson et al., 2007; Cloherty et al., 2010; Cloherty, Crowder, Mustari, & Ibbotson, 2015). One could expect the observed postsaccadic neural enhancement in motion processing areas to result in perceptual enhancement of motion sensitivity. To date, there are no behavioural studies that have specifically looked at whether such enhancement occurs for motion perception in the postsaccadic period in humans, however. Furthermore, a dissociation between OFR and motion perception has been demonstrated in humans, where OFR and motion perception showed opposite sensitivities as a function of complexity of moving textures with natural-like statistics (Simoncini, Perrinet, Montagnini, Mamassian, & Masson, 2012). Although the study did not involve saccades, the results highlight that measures of postsaccadic OFR do not necessarily correspond to measures of postsaccadic motion perception.

Despite evidence for its existence, it is unknown how these cases of postsaccadic enhancement are achieved. Here, we wanted to investigate whether motion sensitivity is enhanced by an antecedent saccade. Based on the evidence of well-correlated postsaccadic neuronal enhancement in motion processing areas and the OFR (Takemura & Kawano, 2006; Ibbotson et al., 2007; Cloherty et al., 2015), and OFR reflecting the behavioural output of centre-surround antagonism and contrast gain control mechanisms of these areas (Barthélemy, Vanzetta, & Masson, 2006; Reynaud, Barthélemy, Masson, & Chavane, 2007), we tested if the postsaccadic enhancement in motion perception can be explained by changes in the balance of centre-surround antagonism in motion processing, where spatial summation is favoured at low contrasts and surround suppression is favoured at high contrasts (Tadin, Lappin, Gilroy, & Blake, 2003). Tadin et al. (2003) showed that perceiving visual motion becomes increasingly more difficult as the size of the motion stimuli increases. The resulting paradoxical elevation of motion direction discrimination thresholds with increasing stimulus size is thought to be a direct perceptual correlate of centre-surround antagonism within cortical area MT/V5 (Tadin, Silvanto, Pascual-Leone, & Battelli, 2011; Liu, Haefner, & Pack, 2016; Schallmo et al., 2018), and is termed spatial suppression. More recently, empirical evidence has emerged suggesting a functional link between spatial suppression and motion segregation (Tadin et al., 2019), where the shift in receptive field organisation from surround suppression to spatial summation was associated with impairments in motion segregation.

In this study, we investigated the influence of an antecedent saccade on motion direction discrimination of a drifting Gabor inside a stationary aperture. We measured minimum duration thresholds for direction discrimination of Gabor stimuli at various sizes, contrasts, and spatial frequencies, presented immediately after saccade offset (no delay) or with a delay of 300 ms (outside the previously reported period of postsaccadic enhancement (Burr et al., 1994; Knoll et al., 2011)). If postsaccadic enhancement is related to the balance of centre-surround antagonism in motion processing areas, then we expect to observe the following: (1) previously observed postsaccadic enhancement of OFR and neural activity in motion processing areas should manifest as better motion discrimination immediately after the saccade compared to outside expected postsaccadic enhancement period, and (2) this observed enhancement in motion sensitivity directly after saccades should be the result of shifts from spatial suppression to more summation, and show corresponding changes in its contrast-dependency in terms of contrast facilitation or inhibition.

## 2 Methods

### 2.1 Participants

15 observers (3 males, 12 females for low spatial frequency; 4 males, 11 females for high spatial frequency) participated in each of the two experiments. All observers were naïve as to the purposes of the experiment and had corrected-to-normal vision better than logMAR 0.10. Stimuli were viewed using their habitual spectacle correction (if needed) and natural pupils. The study followed the Declaration of Helsinki (1964) guidelines and was approved by the institutional ethics committee (proposal number 2015–35 k). All observers gave informed consent prior to participation.

### 2.2 Equipment

Stimuli were presented on a VIEWPixx monitor (VPixx Technologies Inc., Canada) running in M16 mode (16-bit input resolution, 10–12 bit internal resolution) in a darkened room. The display had a spatial and temporal resolution of 1920 × 1080 and 120 Hz, respectively, and was calibrated to ensure a linear gamma correction. The luminance of white, grey, and black pixels was 105.70, 58.33, and 0.21 cd/m^2^, respectively. Observers viewed the stimulus display binocularly at a viewing distance of 60 cm with their heads stabilised by a chin and forehead rest.

Eye movements (single eye) were recorded with a sampling rate of 1000 Hz with an Eyelink 1000 (SR Research Ltd., Ontario, Canada). Experimental software was written in MATLAB (MathWorks, Natick, MA) using the Psychophysics Toolbox (Kleiner et al., 2007) and the Eyelink toolbox (Cornelissen, Peters, & Palmer, 2002). Observers’ responses were given by key press on a standard keyboard.

### 2.3 Stimuli

The stimulus was a drifting Gabor patch: a horizontal sine wave grating surrounded by a two-dimensional Gaussian envelope. The stimulus drifted vertically (up or down) at a speed of 2 deg/sec. We tested two spatial frequency conditions, low and high, that were 1 cyc/deg and 4 cyc/deg respectively, corresponding to temporal frequencies of 2 Hz and 8 Hz. Stimulus size (sigma) was defined as twice the standard deviation of the Gaussian envelope and was either 0.7 or 5 deg. Stimulus contrast was either 2.8 or 92%. Stimuli were presented within a temporal Gaussian envelope, ramping up from zero to peak then down again in contrast. The contrast of the stimulus, therefore, refers to the value of the peak, and the duration of the stimulus is defined as twice the standard deviation of the temporal envelope. Stimuli were presented on a uniform grey background with a constant mean luminance across all conditions that only varied in the sigma and contrast. Stimuli were presented directly after the saccade offset (no delay) or with a delay of 300 ms (delay). This delay period was chosen as the eyes typically fixate a given location for only 200 – 300 ms between saccades in static scenes (e.g. Dorr, Martinetz, Gegenfurtner, & Barth, 2010; Nuthmann, 2017). More importantly, it has previously been shown that postsaccadic enhancement in contrast sensitivity persists only up to 300 ms (Burr et al., 1994; Knoll et al., 2011), and so our delay condition should be, in principle, roughly comparable to a fixation (i.e. no saccade) condition. Therefore, for the low and high spatial frequency conditions, there were eight possible combinations of sigma, contrast levels, and delay condition. Each condition (sigma × contrast) was presented with or without presentation delay in separate blocks of 50 trials each, resulting in 100 trials in total for each block. The block order was counterbalanced across subjects. The two motion directions of the Gabor were equally probable within each block.

### 2.4 Procedure

**Fig. 1:**
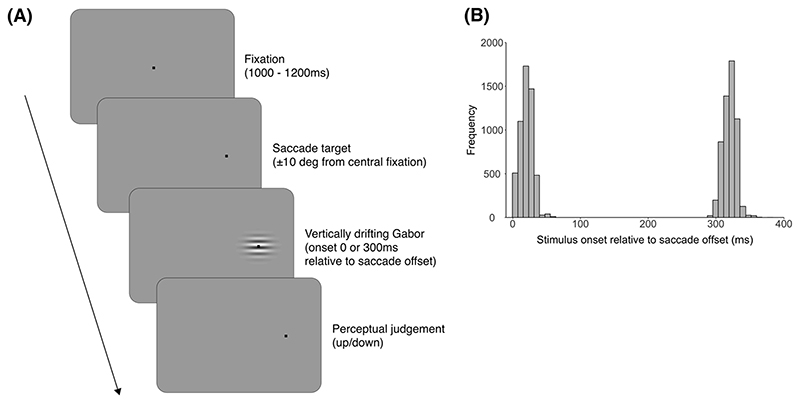
Experimental paradigm. (A) Trial procedure. Stimuli are not drawn to scale. (B) Distributions of stimulus onsets relative to saccade offset for the no delay (0 ms) and delay (300 ms) conditions. The variation in stimulus timing relative to saccade offset within each delay condition was small compared to the timing difference between the no delay and delay conditions.

We measured the minimum duration threshold required for observers to accurately identify the motion direction (Fig. 1). At the beginning of the trial, a fixation spot in the screen centre prompted observers to start the trial by pushing the space bar. After a variable time between 1000 and 1200 ms, the fixation target was extinguished and a new (identical) saccade target appeared 10 deg to the left or right of the screen centre. Observers were required to make a saccade to the new target. For saccade detection online, horizontal velocities of two consecutive samples had to exceed two velocity thresholds (50 and 100 deg/s) (Schütz, Kerzel, & Souto, 2014; Braun, Schütz, & Gegenfurtner, 2017). Once detected, the drifting Gabor stimulus was presented with a fixed delay of 70 ms or 370 ms delay, resulting in average stimulus onset being 21 ± 9 ms and 321 ± 10 ms relative to saccade offset for the no delay and delay conditions, respectively. Observers indicated the perceived motion direction (up or down) by a key press. Feedback was not provided for the correct/incorrectness of the judgement itself but was provided for errors in magnitude, landing, and timing of the saccade relative to the beginning of the stimuli presentation (detailed in section 2.6. Exclusions below).

A QUEST procedure (Watson & Pelli, 1983) was used to determine the minimum duration thresholds for motion discrimination converging on the 82% correct duration threshold. Thresholds were determined from a single block of 50 trials for each condition. The QUEST procedure varied the stimulus duration from trial to trial depending on observers’ performance. The QUEST was updated after each valid trial (see section 2.6. Exclusions for exclusion criteria). Trials were blocked for a single contrast and sigma combination, but presentation condition (delay and no delay) were interleaved within each block. Minimum stimulus duration was set to two frames (16.67 ms).

### 2.5 Data analysis

We quantified the degree of spatial suppression by calculating the suppression index separately for each contrast level. The suppression index (SI) is the log difference of thresholds for large and small diameter stimuli at a given contrast level (Tadin et al., 2006). A positive SI value indicates suppression, while a negative value indicates summation.

Additionally, we quantified the degree of contrast dependency for small and large sigma, by calculating the contrast dependency index separately for each sigma level. The contrast dependency index (CDI) is the log difference of thresholds for high and low contrasts for a given sigma level (Tadin & Lappin, 2005). Negative values of CDI indicate contrast facilitation, while positive values indicate contrast inhibition.

Statistical analyses were performed using R. Two-tailed t-tests were performed for delay and no delay conditions on motion discrimination thresholds, and suppression and contrast dependency indices based on the comparison of size and contrast conditions.

To rule out the potential contribution of retinal modulations from smaller, fixational saccades, we detected trials in which microsaccades occurred during stimulus presentation. Microsaccades were detected using the established algorithm proposed by Engbert and Kliegl (2003) in 2D velocity space using thresholds for peak velocity (6 SD) and minimum duration (6 ms). For the no delay condition, we considered the period 50 ms from saccade offset to stimulus offset. We omitted the first 50 ms after saccade offset as it has been reported that video-based eye trackers do not accurately represent post-saccadic rotations of the eyeball (Kimmel, Mammo, & Newsome, 2012; Nyström, Hooge, & Holmqvist, 2013). For the delay condition, we considered the period from stimulus onset to offset. Overall, we found the percentage of trials where microsaccades occurred were 9% and 2% for the no delay and delay conditions, respectively. When we ran the statistical analyses for all trials, and again by excluding trials in which microsaccades occurred, we found no systematic differences between the results. Therefore, we have included all trials for subsequent analyses.

### 2.6 Exclusions

Eye movement data was analysed offline for trial validity based on the following criteria. Trials were flagged as invalid if eye position during fixation or on saccade landing fell outside a 2 deg radius around the fixation target or the saccade target, respectively. For each trial, saccade amplitudes were calculated using eye positions at saccade onset and offset (detected by the Eyelink 1000 algorithm using a velocity threshold of 22 deg/s and an acceleration threshold of 3800 deg/s^2^) and were flagged as invalid if less than 8 deg. For the no delay condition, trials in which more than 20% of the motion stimulus duration fell before the detected saccade offset (i.e. during the saccade when vision is suppressed) were excluded. For consistency in the delay condition, we excluded trials if more than 20% of the motion duration occurred before 300 ms from saccade offset. These exclusion criteria resulted in the exclusion of a small proportion of trials across observers. For low spatial frequency stimuli, 6% (180 of total 3000 trials) and 4% (136 out of 3000 trials) of trials were excluded for the no delay and delay conditions, respectively. For high spatial frequency, 7% (209 of 3000 trials) and 6% (179 of 3000 trials) of trials were excluded for the no delay and delay conditions, respectively.

## 3 Results

We measured motion discrimination for stimuli differing in size, contrast, and spatial frequency at two different points in time: directly after the offset of a saccade or 300 ms later. We first report raw motion discrimination thresholds and later on suppression and contrast dependency indices based on the comparison of size and contrast conditions.

### 3.1 Motion discrimination duration thresholds

Motion discrimination thresholds of low and high contrast, and small and large sigma stimuli were compared for the delay and no delay condition. A lower duration threshold immediately after the saccade would indicate postsaccadic enhancement of motion sensitivity.

**Fig. 2:**
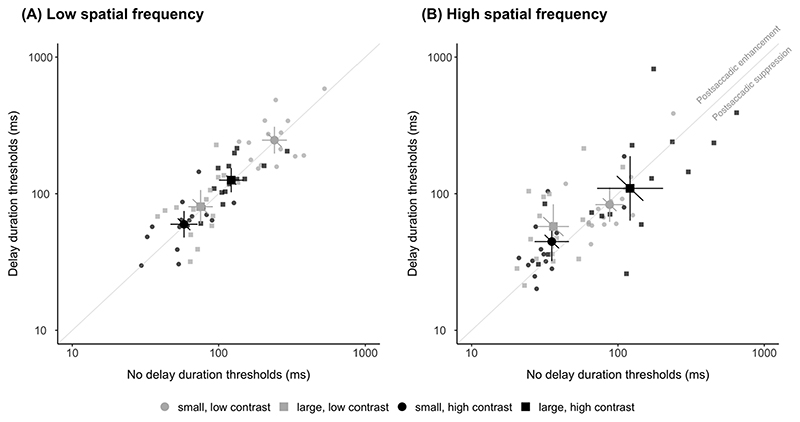
Minimum duration thresholds for delay plotted against no delay condition for (A) low spatial frequency and (B) high spatial frequency Gabor stimuli. Low contrast (grey symbols) and high contrast (black symbols) for small (circles) and large (square) stimuli. Small symbols indicate data from individual observers; large symbols indicate the mean across observers. Error bars denote 95% confidence intervals. As noted in (B), points above the unity line indicate postsaccadic enhancement of motion.

For low spatial frequency stimuli (Fig. 2A), we found motion duration thresholds (mean ± SEM) immediately after saccade offset (no delay condition) were no different to those that are presented with a 300 ms delay (delay condition) for all combinations of sigma and contrast conditions (paired t-tests, all *ps* > 0.30). For small stimuli, duration thresholds for no delay (256.2 ± 26.0 ms) and delay (268.4 ± 32.6 ms) were no different (*t*(14) = −0.430, *p* = 0.67) at low contrast. Similarly, for high contrast, the no delay (62.2 ± 6.4 ms) and delay (64.5 ± 7.2 ms) conditions did not differ (*t*(14) = −0.339, *p* = 0.74). For large stimuli, there was no difference in thresholds at low contrast (*t*(14) = −1.047, *p* = 0.31) between no delay (79.5 ± 6.7) and delay (90.8 ± 12.7 ms). Similarly, there was no difference in thresholds for high contrast (*t*(14) = −0.311, *p* = 0.76) between no delay (130.0 ± 14.3 ms) and delay (133.6 ±12.2 ms). Group averages of measured duration thresholds for each combination of sigma and contrast conditions all lie on the unity line of the scatter plot, suggesting motion sensitivity was unaltered by the antecedent saccade for low spatial frequency stimuli.

For high spatial frequency stimuli (Fig. 2B), results differed between conditions. For small stimuli at low contrast, there was no difference (*t* (14) = −0.273, *p* = 0.79) between the no delay (95.3 ± 12.0 ms) and the delay (98.8 ± 21.6 ms) condition. At high contrast, there was a trend for lower duration thresholds with no delay (40.7 ± 7.4 ms) compared to delay (54.3 ± 11.2 ms), evident by group means falling above the unity line. However, this failed to reach significance (*t*(14) = −1.866, *p* = 0.08). For large stimuli, duration thresholds for low contrast stimuli were significantly lower with the no delay (40.2 ± 5.8 ms) compared to the delay (72.1 ± 14.1) condition (*t*(14) = −2.721, *p* = 0.02). Lastly, there was no difference at high contrast between the no delay (179.5 ± 44.6 ms) and delay (175.6 ± 53.1 ms) condition (*t*(14) = 0.073, *p* = 0.94). These results demonstrate postsaccadic enhancement of motion sensitivity for high-spatial frequencies, with significantly lower thresholds directly after the saccade for large, low contrast stimuli, and a similar trend for small, high contrast stimuli.

### 3.2 Suppression index

The degree of spatial suppression was quantified by calculating the suppression index as the log difference between thresholds for small and large stimuli at each contrast level. Changes in SI immediately after the saccade, depending on the direction of change, would indicate a shift in the balance of spatial suppression or summation with more positive and negative indices, respectively.

**Fig. 3:**
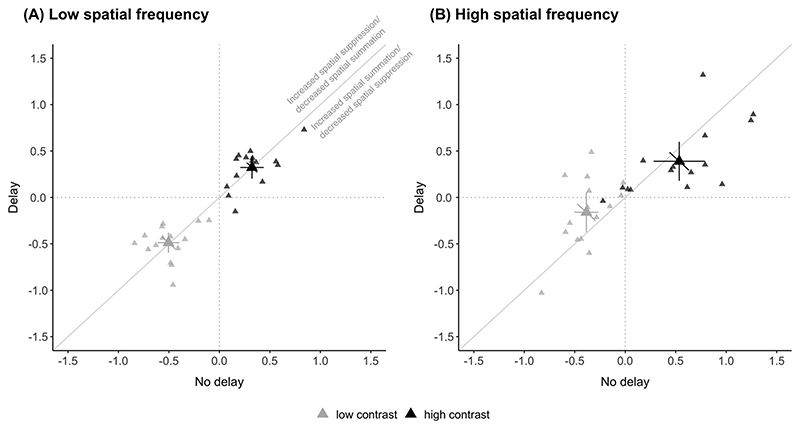
Suppression index for low (gray) and high (black) contrast conditions for (A) low spatial frequency and (B) high spatial frequency Gabor stimuli. Positive values indicate spatial suppression; negative values indicate spatial summation. As noted in (A), values above the diagonal indicate increased spatial suppression (or less spatial summation) directly after the saccade. Values below the diagonal indicate decreased spatial suppression (or increased spatial summation) directly after the saccade. SI is calculated from taking the log difference of large and small diameter thresholds. Note that these values are derived from corresponding black and grey points in the minimum duration threshold plot in Fig. 2. Other conventions are the same as in Fig. 2.

For low spatial frequency (Fig. 3A), there were no differences between the delay and no delay conditions in the SI for low and high contrast stimuli. Calculated SI (mean ± SEM) for low contrast for no delay (−0.51 ± 0.05) and delay (−0.49 ± 0.05) did not differ (*t*(14) = −0.280, *p* = 0.78). Similarly, for high contrast, SI for no delay (0.32 ± 0.05) and delay (0.32 ± 0.06) did not differ (*t*(14) = −0.001, *p* = 0.99). Group averages of SI fall on the unity line, and the results suggest that the balance of spatial suppression and summation were unaltered by the antecedent saccade for low spatial frequency stimuli at both low and high contrast levels.

For high spatial frequency (Fig. 3B), SI for low contrast were significantly different for no delay (−0.39 ± 0.06) compared to the delay (−0.16 ± 0.10) condition (*t*(14) = −2.66, *p* = 0.02). For high contrast, we found a trend towards a more positive SI for the no delay (0.54 ± 0.12) condition relative to the delay (0.39 ± 0.10) condition – group means falling below the unity line – although the difference was not significant (*t*(14) = 1.61, *p* = 0.13). Immediately after saccades, the SI was more positive in value compared to the delay condition for high contrast stimuli, although not statistically significant, suggesting a trend towards increased spatial suppression at high contrasts. Furthermore, our findings that the SI for the no delay condition was more negative compared to the delay condition for low contrast stimuli, indicates increased summation at low contrasts immediately after saccades.

### 3.3 Contrast dependency index

The degree of contrast facilitation or inhibition was quantified by calculating the contrast dependency index as the log difference between thresholds for high and low contrast stimuli at each sigma level. Changes in CDI reflect changes in the asymmetric interactions between excitatory centre and inhibitory surround mechanisms. Therefore, changes in CDI immediately after the saccade, depending on the direction of change relative to the delay condition, would indicate contrast inhibition or facilitation with more negative and positive indices, respectively.

**Fig. 4:**
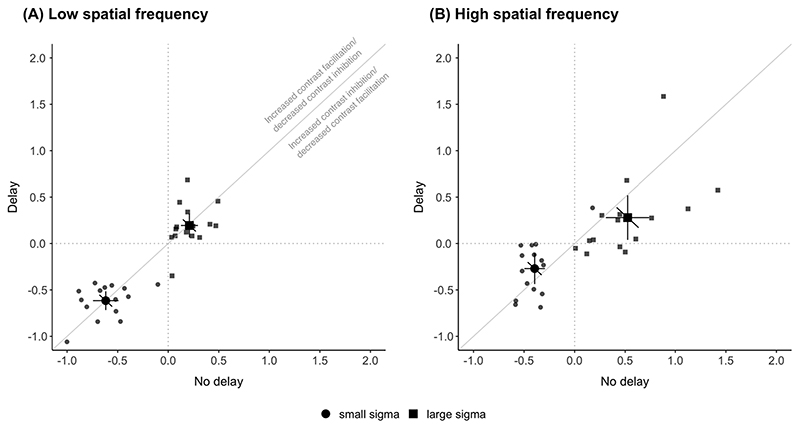
Contrast dependency index for small (circles) and large (squares) diameter condition for (A) low spatial frequency and (B) high spatial frequency Gabor stimuli. Positive values indicate contrast inhibition; negative values indicate contrast facilitation. As noted in (A), values above the diagonal indicate increased contrast facilitation (or decreased contrast inhibition) directly after the saccade. Values below the diagonal indicate increased contrast inhibition (or decreased contrast facilitation) directly after the saccade. CDI is calculated from taking the log difference of high and low contrast thresholds. Note that these values are derived from corresponding circle and square symbol points in the minimum duration threshold plot in Fig. 2. Other conventions are the same as in Fig. 2.

For low spatial frequency (Fig. 4A), there were no differences in the CDI between the delay and no delay conditions. The CDI value (mean ± SEM) for small sigma for no delay (−0.62 ± 0.06) and delay (0.62 ± 0.05) were not different (*t*(14) = −0.040, *p* = 0.97). For large sigma, there were no differences in CDI between the no delay (0.21 ± 0.04) and delay (0.19 ± 0.06) conditions (*t*(14) = −0.252, *p* = 0.80). Group averages of CDI fall on the unity line suggesting that there were no contrast-dependent changes associated with the antecedent saccade, as would be expected from the absence of any postsaccadic modulation of motion duration thresholds for low spatial frequency stimuli (Fig. 2A).

For high spatial frequency (Fig. 4B), CDI (mean ± SEM) values for small sigma were more negative for no delay (−0.40 ± 0.05) compared to delay (−0.27 ± 0.08) conditions, although this difference was not significant (*t*(14) = −1.943, *p* = 0.07). For large sigma, calculated CDIs were significantly different between the no delay (0.53 ± 0.10) and delay (0.28 ± 0.11) conditions (*t*(14) = −2.413, *p* = 0.03). The results show increased contrast inhibition for large stimuli and a trend towards increased contrast facilitation for small stimuli.

## 4 Discussion

In our study, we found evidence of selective postsaccadic enhancement of motion sensitivity for high, but not for low spatial frequency stimuli. Lower minimum motion duration thresholds were observed immediately after the saccade, consistent with our expectations based on psychophysical, oculomotor, and physiological studies demonstrating modified processing and enhanced performance immediately after saccades. We quantified the degree of change in spatial suppression and contrast dependency by calculating suppression and contrast dependency indices and compared the differences between whether motion was presented within or outside the previously reported period of postsaccadic enhancement in our no delay and delay conditions, respectively. These indices showed that the balance of spatial suppression and summation was altered immediately after a saccade, compared to a delay of 300 ms relative to saccade offset. Overall, our results showed that postsaccadic enhancement of motion sensitivity is associated with changes in spatial interactions between excitatory centre and inhibitory surround, and in contrast facilitation and inhibition.

Our results demonstrate a functional benefit of a dynamic balance in receptive field organisation between spatial suppression and summation. Under conditions of low visibility – i.e., low contrast and/or small stimuli – we observed a shift towards spatial summation and increased contrast facilitation. Conversely, under conditions of high visibility – i. e., high contrast and/or large stimuli – a shift towards spatial suppression and increased contrast inhibition occurred immediately after saccades. To our knowledge, our study is the first to show a psychophysical postsaccadic enhancement of motion sensitivity in humans. Our study fills a gap in the current literature, where evidence for postsaccadic enhancement in humans is limited to studies on contrast sensitivity for stationary stimuli (Burr et al., 1994; Diamond, Ross, & Morrone, 2000; Knoll et al., 2011; Dorr & Bex, 2013), while, evidence of postsaccadic changes and enhancement in neuronal responses and motion sensitivity have been limited to studies of non-human primates (Ibbotson et al., 2007; Ibbotson et al., 2008; Bremmer et al., 2009), leaving a gap in our knowledge about whether these findings also translate to enhanced postsaccadic motion perception in humans.

Our study is distinct from those that have studied postsaccadic enhancement of contrast sensitivity (Burr et al., 1994; Knoll et al., 2011; Dorr & Bex, 2013), as our task required observers to perceive motion to discriminate its direction; noticing the flash or modulation of contrast would have been insufficient to solve the task. We found, for motion stimuli of high spatial frequency, the magnitude of postsaccadic enhancement was 0.25 and 0.13 log units for low contrast large, and high contrast small stimuli, respectively. These magnitudes are similar, albeit slightly smaller than those reported previously for chromatic contrast sensitivity, which was in the order of around 0.3 log units (Burr et al., 1994). Similar magnitudes of postsaccadic enhancement for chromatic contrast sensitivity were observed in the study of Knoll et al. (2011), with similar temporal profiles of enhancement being observed under both active and passive (displacement of visual display simulating retinal motion caused by a saccade during fixation) conditions.

### 4.1 Postsaccadic enhancement – active vs. passive mechanisms?

A long-standing question is whether modulations of perception around the time of eye movements are passive phenomena due to the retinal consequences of the eye movements or an active mechanism, for instance an extra-retinal modulation of visual processing (for reviews see Matin, 1974; Ross, Morrone, Goldberg, & Burr, 2001; Binda & Morrone, 2018).

Most of that research has concentrated on saccadic suppression, where passive as well as active contributions have been considered: First, retinal smear due to the fast retinal image motion during the saccade (e.g. Burr & Ross, 1982; but see Castet & Masson, 2000), second, masking by the clear and highly reliable visual input before and after the saccade (Matin, Clymer, & Matin, 1972; Campbell & Wurtz, 1978; Castet, Jeanjean, & Masson, 2002; Duyck, Collins, & Wexler, 2016), and third, an active, extra-retinal mechanism downregulating contrast gain (e.g. Zuber & Stark, 1966; Volkmann et al., 1968, 1978; Burr et al., 1994; Diamond et al., 2000). Potentially relevant for postsaccadic enhancement, the active mechanism has also been associated with a reduced duration of saccadic suppression (Diamond et al., 2000; Idrees, Baumann, Franke, Münch, & Hafed, 2020) that appears to be longer in simulated than in real saccades.

With respect to postsaccadic enhancement of contrast sensitivity, there are inconsistent findings. While Knoll et al. (2011) observed similar enhancement of chromatic contrast sensitivity for real and simulated saccades on a homogeneous background, Dorr and Bex (2013) found postsaccadic enhancement of luminance sensitivity only for real but not for simulated saccades on a naturalistic background. Both, the background (homogeneous vs. naturalistic) and the type of mechanism (color vs. luminance) might contribute to the discrepant results. The enhancement of chromatic contrast sensitivity during smooth pursuit has been linked to an active mechanism because it starts well before the onset of the smooth pursuit eye movement (Schütz, Braun, Kerzel, & Gegenfurtner, 2008).

With respect to oculomotor and neurophysiological enhancement effects, there are also diverging results. The enhancement of the OFR seems to primarily be a passive effect (Kawano & Miles, 1986), because it is strongly reduced when the background does not elicit a strong retinal motion during the saccade and because it can be triggered by a passive movement of the visual background during fixation. In contrast, the enhancement of motion processing in area MST is only present in real, but not in simulated saccades (Cloherty et al., 2015).

We consider whether our findings of selective postsaccadic changes in spatial suppression and summation, and contrast gain related to motion perception can add to this discussion. Specifically, whether postsaccadic changes result from an active modulation of visual processing, from the passive consequences of retinal image motion during the saccade, or from the passive consequences of changes in post-saccadic oculomotor behaviour (i.e. smaller, fixational saccades). A few properties of the experimental paradigm are in favour of an active process. First, we used a blank background, such that only the screen borders produced intrasaccadic motion signals. Second, our stimulus was shown after saccade offset, such that the retinal input during stimulus presentation was comparable in the delay and no delay conditions. However, it is possible that after the primary saccade, participants executed additional small saccades in one delay condition more than the other and that the resulting modulations of retinal input contributed to our results. Rucci and colleagues have suggested that small saccades during fixation may lead to improvements in visual sensitivity by redistributing the spatiotemporal power of stationary stimuli, and that microsaccades and smaller saccades preserve power at low spatial frequencies (Mostofi et al., 2020; Mostofi, Boi, & Rucci, 2016). Nevertheless, the fact that we found the same pattern of results when we excluded trials in which microsaccades occurred, argues against the possibility that our results are a passive consequence of smaller, secondary saccades. Furthermore, a tentative physiological correlate would be in favour of an active process as well. As it is widely accepted that spatial suppression is a behavioural correlate of centre-surround antagonism within cortical area MT (Churan, Khawaja, Tsui, & Pack, 2008; Tadin et al., 2011; Liu et al., 2016; Schallmo et al., 2018); that we find selective postsaccadic changes in spatial suppression and contrast gain related to enhanced motion sensitivity suggests that the saccade-related, active mechanisms implicated in the neuronal enhancement in areas MT/MST may be involved. However, these ideas are speculative in the absence of a passive control condition in the current study and remains an unanswered question worth exploring in the future.

### 4.2 Dynamic nature of centre-surround antagonism and optimal size

Our results suggest that the observed enhancement in motion sensitivity resulted from the dynamic regulation of spatial suppression and summation, and contrast facilitation and inhibition. At low contrast, we observed an increase in spatial summation in the order of 0.4 log units immediately after saccades compared to the delay condition. At high contrast, spatial suppression was 0.14 log units higher immediately after saccades, compared to the delay condition. These values are in the range of other modulations of spatial summation reported in the literature. Tadin et al. (2019) found an increase in spatial suppression strength that was direction-specific following training in a motion segregation task confined to one motion direction – the magnitude of difference being around 0.6 log units higher for the trained compared to the untrained direction. In studies looking at the effect of aging on spatial suppression, differences of around 0.3–0.5 log units in suppression indices between older and younger adults have been reported, with older adults exhibiting less suppression compared to younger adults (Pitchaimuthu et al., 2017; Tadin et al., 2019).

These findings provide evidence for the dynamic nature of centre-surround antagonism, specifically the spatial properties of receptive field being dependent on visual context. The optimal size for perceiving motion is posited to indicate the size at which the inhibitory surround dominates over excitatory center mechanisms, with optimal size shown to decrease approximately two-fold with increasing contrast (Tadin & Lappin, 2005). These findings agree with previous reports of V1 receptive field size decreasing with contrast, resulting in receptive field size changes between 0.3 and 0.6 log units (Kapadia, Westheimer, & Gilbert, 1999; Sceniak, Ringach, Hawken, & Shapley, 1999; Cavanaugh, Bair, & Movshon, 2002). Similarly in area MT, centre-surround antagonism observed at high contrast has been shown to substantially weaken or even disappear at low contrast levels (Pack, Hunter, & Born, 2005).

While previous studies demonstrate the contrast dependency and dynamic nature of spatial suppression both within and between subject populations, our study adds to the literature by providing evidence for the dynamic modulation of these factors around eye movements.

### 4.3 Spatial tuning of enhancement

In this study, postsaccadic enhancement of motion sensitivity was limited to high spatial frequency stimuli. On the one hand, this is consistent with several other cases of perceptual modulations that are selective for specific spatial frequency bands. The enhancement of contrast sensitivity during smooth pursuit eye movements is selective for high spatial frequencies and color (Schütz et al., 2008, 2009) and saccadic suppression is selective for low spatial frequencies (Volkmann, Riggs, White, & Moore, 1978; Burr, Holt, Johnstone, & Ross, 1982; Burr, Morrone, & Ross, 1994; Braun, Schütz, & Gegenfurtner, 2017). Similar to well-established suppression associated with larger saccades, a recent study by Scholes et al. (2018) showed greatest suppression for low spatial frequencies during microsaccades. However, an unexpected finding was that there was enhancement of visual sensitivity (contrast detection) at higher spatial frequencies, peaking for stimuli of 1–2 cycles/deg around 100–200 ms after microsaccade onset. Their results demonstrated that changes in spatial sensitivity around the time of microsaccades resulted from differences in tuning of perisaccadic suppression and postsaccadic facilitation (Scholes et al., 2018).

Our low (1 cycle/deg) and high (4 cycles/deg) spatial frequency conditions were of the same speed, corresponding to temporal frequencies of 2 Hz and 8 Hz, respectively. Hence, it is not possible to distinguish whether the postsaccadic enhancement in our results was specific to spatial frequency or temporal frequency. In area MT, tuning properties of MT neurons have been observed to shift with contrast. Responses are well described by separable tuning (independent tuning for spatial and temporal frequency) when contrast is low, but move towards speed-like tuning (same speed preference irrespective of spatial frequency) when contrast is high (Priebe, Cassanello, & Lisberger, 2003). These results demonstrate the effect of contrast on the interaction of sensitivities to spatial and temporal frequency (Priebe et al., 2003; Priebe, 2006) and suggest that effects originating from motion sensitive areas could be specific in terms of spatial frequency or speed.

### 4.4 Contrast dependency and ocular following response

It is well established that the early phase of OFR is enhanced following a saccade, in both speed and latency (Kawano & Miles, 1986), and that the time course of this enhancement is similar to the time course of enhanced responses of area MT/MST after a saccade (Cloherty et al., 2010; Ibbotson et al., 2007; Takemura & Kawano, 2006). Importantly, it has been shown that neuronal enhancement is not directly driven by saccade-related spiking activity but occurs afferently to MT and is influenced by the speed tuning of cells (Ibbotson et al., 2007). Our study provides evidence in humans that motion sensitivity is enhanced after the saccade, as would be expected based on previous reports from non-human primate studies showing postsaccadic neuronal enhancement in motion processing areas and postsaccadic enhancement of OFR (Cloherty et al., 2015; Ibbotson et al., 2007). OFR has also been studied in humans (Masson et al., 2000; Masson et al., 2001; Masson & Castet, 2002; Quaia, Sheliga, Fitzgibbon, & Optican, 2012; Simoncini et al., 2012). Our results suggest that in humans, motion perception is similarly enhanced by saccades as OFR (Gellman, Carl, & Miles, 1990).

Contrast gain control, spatial summation, and surround dynamics have also been investigated in detail at the behavioural level using OFR (Barthélemy et al., 2006; Reynaud et al., 2007). OFR exhibits many properties attributed to the earliest stages of motion detection and integration, where contrast dependent effects of spatial summation area (optimal size) of macaque V1 and MT (Kapadia et al., 1999; Sceniak et al., 1999; Cavanaugh et al., 2002; Pack et al., 2005) lead to the observed changes in OFR. It has been observed, that increasing contrast from 2.5 to 80% results in a four-fold increase in response amplitude over the first 100 ms of the OFR in the macaque (Reynaud et al., 2007). In humans, Barthélemy et al. (2006) showed a larger spatial summation area for low contrast compared to that of medium and high contrast stimuli, as would be expected from contrast-dependent changes in receptive field sizes in V1 (Kapadia et al., 1999; Sceniak et al., 1999; Cavanaugh et al., 2002) and MT (Pack et al., 2005). Overall, the results demonstrate that increasing contrast leads to both enhancement of response amplitude and reduction in the width of the spatial integration area for ocular following.

Whilst our study does not allow us to point to a specific mechanism or mechanisms driving the perceptual enhancement of motion sensitivity observed in our study, existing mechanisms such as the dynamic nature of centre-surround antagonism and optimal size, and tuning properties of neurons involved in motion processing, may be involved. These may work in concert or in isolation to increase the visual responsivity and sensitivity after a saccade. These mechanisms show dynamic modulations in response to contrast and provide a potential explanation for how increased motion sensitivity in humans may arise from increased spatial and temporal integration, and changes in contrast gain immediately after saccades.

## 5 Conclusions

To conclude, we found selective enhancement of motion perception immediately after saccades for high spatial frequency stimuli, consistent with previously reported selective postsaccadic enhancement of contrast sensitivity for flashed high spatial frequency stimuli. The observed enhancement was associated with changes in spatial summation and suppression, as well as contrast facilitation and inhibition, suggesting that motion processing is augmented to maximise visual perception immediately after saccades. These results highlight that spatial and contrast properties of the underlying neural mechanisms for motion processing can be affected by an antecedent saccade for high spatial frequency stimuli.
